# Understanding data collection strategies for the ethical inclusion of older adults with disabilities in transitional care research: A scoping review protocol

**DOI:** 10.1371/journal.pone.0293329

**Published:** 2023-10-20

**Authors:** Kristina M. Kokorelias, Reham Abdelhalim, Marianne Saragosa, Michelle L. A. Nelson, Hardeep K. Singh, Sarah E. P. Munce

**Affiliations:** 1 Division of Geriatric Medicine, Department of Medicine, Sinai Health System and University Health Network, Toronto, Toronto, Ontario, Canada; 2 Department of Occupational Science & Occupational Therapy, Temerty Faculty of Medicine, University of Toronto, Toronto, Ontario, Canada; 3 National Institute on Ageing, Toronto Metropolitan University, Toronto, Ontario, Canada; 4 Burlington OHT, Burlington, Ontario, Canada; 5 Joseph Brant Hospital, Burlington, Canada; 6 KITE Toronto Rehabilitation Institute-University Health Network, Toronto, Ontario, Canada; 7 Lunenfeld-Tanenbaum Research Institute, Sinai Health, Toronto, Ontario, Canada; 8 Institute of Health Policy, Management and Evaluation, University of Toronto, Toronto, Canada; 9 Rehabilitation Sciences Institute, Temerty Faculty of Medicine, University of Toronto, Toronto, Canada; University of Saskatchewan, CANADA

## Abstract

**Introduction:**

A growing body of evidence suggests that older adults are particularly vulnerable to poor care as they transition across care environments. Thus, they require transitional care services as they transition across healthcare settings. To help make intervention research meaningful to the older adults the intervention aims to serve, many researchers aim to study their experiences, by actively involving them in research processes. However, collecting data from older adults with various forms of disability often assumes that the research methods selected are appropriate for them. This scoping review will map the evidence on research methods to collect data from older adults with disabilities within the transitional care literature.

**Methods:**

The proposed scoping review follows the framework originally described by the Joanna Briggs Institute (JBI) Manual: (1) developing a search strategy, (2) evidence screening and selection, (3) data extraction; and (4) analysis. We will include studies identified through a comprehensive search of peer-reviewed and empirical literature reporting on research methods used to elicit the experiences of older adults with disabilities in transitional care interventions. In addition, we will search the reference lists of included studies. The findings of this review will be narratively synthesized. The Preferred Reporting Items for Systematic reviews and Meta-Analyses extension for Scoping Reviews will guide the reporting of the methods and results.

**Discussion:**

The overarching goal of this study is to develop strategies to assist the research community in increasing the inclusion of older adults with disabilities in transitional care research. The findings of this review will highlight recommendations for research to inform data collection within future intervention research for older adults with disabilities. Study findings will be disseminated via a publication and presentations.

## Introduction

High-quality care is especially important for older adults who may become disabled (e.g., due to cognitive impairments or physical disabilities), as well as for their family caregivers, as they transition within and across care settings (e.g., acute care to rehabilitation) [1–4] and health care providers. These transitions in care often result in discontinuity of services, unmet patient and caregiver needs, poor satisfaction with care and increased risk of (re)hospitalization [5–7]. This poor quality of care can be partially explained by problems with information exchange and a lack of multidisciplinary collaboration with patients and healthcare providers reporting problems during discharge [8–11].

To help older adults overcome these challenges, many health care systems have developed transitional care interventions [12–14] designed to improve the continuity of high-quality care [15]. Numerous scoping, systematic reviews and meta-analyses have been conducted on the effectiveness of these interventions [6, 13, 15–17]. Exploring the experiences and satisfaction of older patients has become an increasingly valuable means to evaluate healthcare interventions and the performance of the care providers and health care systems [18–24]. Moreover, if transitional care interventions are to be the solution for improving older adults’ well-being across the care continuum, it is necessary to comprehend the complexity of causes that influence older adults’ experiences of care [25–29]. Thus, collecting data from older adults with different backgrounds and forms of disability could help provide more effective and sustainable transitional care interventions [30].

Experiences of patients during transitions in care and transitional interventions can and have be collected through various means. This includes in-depth qualitative interviews (e.g., [31–33]) and/or focus groups [34, 35], observations [36], and surveys to capture patient-reported outcome and experience measures (PROMs and PREMs) [37]. However, these strategies may ignore the wide number of older adults with disabilities [38], who may struggle to converse (e.g., aphasia) [39], struggle with hearing interviewers [40], hold a pen (e.g., skeletal diseases) [41], or lack cognitive capacity [42]. Compassionate and fair research practices could consider various data collection approaches that would facilitate the inclusion of older adults living with disabilities. Thus, there is a need for improved and diverse strategies in the research context to support appropriate opportunities for the wider inclusion of older adults in transitional care interventions [27, 43]. Driven by the purpose of exploring strategies to support the compassionate involvement of older adults with various physical, mental and cognitive disabilities in research, this proposed study aims to present a summary and map of the existing research methods being used within the transitional care research as an encouragement to future research endeavors.

## Methods

We will conduct a scoping review to examine the transitional care literature for older adults living with disabilities to better understand data collection methods applicable to older adults with age-related disabilities. A scoping review methodology was selected to allow us to explore the broad research topic and obtain knowledge from across study designs [44, 45]. We will follow the scoping review methods outlined by the Joanna Briggs Institute (JBI) Manual for scoping reviews, including a framework for conducting scoping review studies [46]. This framework will include the following steps: (1) developing a search strategy, (2) evidence screening and selection, (3) data extraction; and (4) analysis [46]. The reporting of the review will be informed by the Preferred Reporting Items for Systematic Reviews and Meta-analysis for Protocols (PRISMA-P) [47] and the PRISMA extension for scoping reviews (PRISMA-ScR) [48]. All research team members have reviewed and approved the draft protocol and registered with Open Science Framework [Blinded for Review].

The research questions were developed and refined by the research team. This review aims to learn from various data collection methods within the transitional care literature that include older adults with diverse disabilities to inform future methodological considerations in forthcoming research. This proposed review will address the following research questions and sub-questions:

1. What is the extent, range and nature of research methods used within the transitional care research that have included older adults living with disabilities?
  - How do researchers accommodate sensory, physical, mental and cognitive disabilities in their recruitment and data collection methods?
2. What are the characteristics of the older adults living with disabilities that have been included in transitional care interventions? literature?
  - What older adults are being excluded from research on their experiences within the transitional care intervention literature as a result of their disabilities?

### Framework

The framework of Sex- and Gender-Based Analysis Plus (SGBA+) [49] was used as a starting point for conceptualizing and developing this research protocol. This framework has guided other literature reviews [27, 50, 51] and evaluations of health interventions [52, 53] by using intersectional lenses [54, 55] to examine characteristics of participants and samples within research processes [49]. This framework considers both biological sex (sex), the social construct of gender (gender), as well as other intersectional characteristics including ethnicity, income, age, race, education, and sexual orientation [49].

### Stage 1: Developing a search strategy

The search strategy will be created and drafted OVID Medline by an Information Specialist and Health Science librarian (EP), in consultation with the primary and senior author (KMK and SEPM). Subject headings and text words related to the following concepts will be included in the search: ‘older adults’ ‘disability’ ‘transitions in care’ ‘healthcare continuum’ and ‘methods’. To ensure a breadth of understanding, we will conceptualize disability as per the International Classification of Functioning Disability and Health (ICF) as an “umbrella term for impairments, activity limitations or participation restrictions” [56]. During the search development process, we will limit the search to English. We will limit to papers published from the past two decades (i.e., 2003 onwards) to capture the most up-to-date literature to inform future research due to resource constraints. No design limitations will be imposed.

Once the entire research team approves the final search strategy, the strategy will undergo peer-review using the Peer Review of Electronic Search Strategies (PRESS) Statement [57]. Peer-reviewing the strategy will help to enhance the comprehensiveness of the search [57]. Once the search is finalized, the search will then be translated to OVID Embase, Social Work abstracts, PEDroPhysiotherapy Evidence Database, OVID PsycINFO, EBSCO CINAHL, ERIC, the Cochrane Library, Scopus and Global Index Medicus and run by the Information Specialist and Health Science librarian. Search results will be imported into an Endnote library by the information specialist for reference management and articles will be deduplicated following the Bramer method [58, 59]. To ensure a comprehensive search, we will search for articles not captured within the search, we will hand-search reference lists of included articles and relevant reviews [44]. We will also hand-search for the full-text articles of relevant conference abstracts and study protocols.

### Stage 2: Evidence screening and selection

The deduplicated studies will be imported to Covidence, to help manage screening (i.e., title/abstract screening and full-text article screening) [60, 61]. The Population, Concept, Context (PCC mnemonic) criteria [62] helped to inform the inclusion criteria outlined in Table 1.

**Table 1:** Inclusion and exclusion criteria.

Criteria	Inclusion	Exclusion
Population:	Eligible studies will include older adults living with any type of disability regardless of time of onset (i.e., acquired during old age or developed early in life). Older adults are defined as adults aged 65 years or older. Disability will include “limitations in capacities which are needed to participate in daily life” and incorporate “impairment in body structures or functions”, “capacity limitations”, “environment”, and “participation” [56] p. 125. In order to be included, >1 older adult with disability will need have been involved in data collection as a participant.	Populations other than older adults with disabilities. Older adults with disabilities who were not involved in data collection and a participant in the study.
Concept:	Studies that describe an intervention (i.e., policies, program and practices) to assist an older adult with the transition from one healthcare setting to another (e.g., from acute to in-patient rehabilitation). Any comparator is relevant for inclusion (e.g., studies comparing a transitional intervention to standard practice). In addition, studies without a comparator are eligible for inclusion (e.g., studies examining experiences with an intervention)	Studies that focus on transitions from hospital to home, where care at home will not be provided by a registered healthcare (i.e., no follow up care being done at home) Studies that focus on a interventions that have not yet been implemented. Studies that report on pharmacological interventions.
Context:	Studies that identify and/or employ strategies for engaging older adults with disabilities as participants will be included.	Protocol papers or papers refining or developing conceptual models, methods and frameworks to guide data collection from older adults with disabilities will be excluded.
Study designs:	All study designs using empirical data collection (i.e., qualitative, quantitative or mixed method methodologies) will be eligible for inclusion, except for case reports.	Non-empirical literature and relevant grey literature (e.g., conference abstracts, theses and dissertations). We will search for the full-text articles of conference abstracts and study protocols that fulfil our eligibility criteria during a hand-search.
Time periods:	To increase feasibility, we will restrict inclusion to the past 20 years.	
Setting:	Studies in any healthcare setting or country will be considered for inclusion.	
Language:	Only full-text papers written in English will be considered for inclusion.	Literature not available in full-text in English

At least two reviewers will independently review articles using the above eligibility criteria for level 1 (i.e., title and abstract) and level 2 (i.e., full text) screening. To ensure high inter-rater reliability, prior to starting the title/abstract screening process (level 1 screening), the inclusion criteria will be tested on a random sample of 10% of the articles. We will proceed with independent screening (i.e., each reviewer reviewing articles on their own) when there is a minimum inter-rater agreement of >75% agreement across the team. If we do not achieve this with the 10% pilot screening, the inclusion and exclusion criteria will be modified to be clearer, and the pilot will be repeated with another 10% of titles and abstracts [63]. Following an appropriate understanding of the inclusion and exclusion criteria and inter-rater agreement, the remaining title and abstract screening and full-text screening will be conducted by two reviewers independently, in duplicate. Conflicts at all stages will be resolved by the senior responsible author as the third reviewer (SEPM). Where there is uncertainty, conflicts will be resolved through team discussions during meetings. The screeners will meet bi-weekly throughout the screening process to discuss their initial perceptions of the data [63].

### Stage 3: Data extraction

As with screening, data extraction will be conducted by two reviewers. Data extraction will be an iterative process, with the final categories only being determined as the authors become more familiar with the data [63]. However, it is anticipated that data will be extracted on study characteristics (e.g., study design, country of the corresponding author, method for data collection, method for recruitment) and population characteristics (e.g., number of participants, type of disability, characteristics of the intervention, country, health system settings, the objective of data collection, resources required to accommodate disabilities, theory or framework used to inform the data collection). We will also categorize participants according to the SGBA+ (e.g., sex, gender, and other identity constructs) [49].

Data abstraction will be facilitated using a customizable form in Covidence. First, the two reviewers will extract data independently from a random sample of five included studies. If there is >75% agreement across the two extraction forms, the two reviewers will abstract data on 50% of the training articles (i.e., not in duplicate, with each doing an equal amount). If a poor agreement is found, the data abstraction form will be clarified, and the two reviewers will abstract data independently, with conflicts resolved by a third reviewer. In both cases, the data will be checked by the senior responsible author (SEPM).

As consistent with the Joanna Briggs Institute Manual, appraisal for risk of bias and quality of the studies will not be performed [45, 64].

### Stage 4: Data analysis

Results will be summarized quantitatively (using numerical frequencies) and qualitatively (drawing on content analysis methods [65, 66]), as recommended for scoping reviews [67]. If possible, we will stratify results by type of disability experienced by the older adult participants and transition in care settings (e.g., type of transition, i.e., healthcare settings involved in the transition). Specifically, we will perform line-by-line coding to inform the development of descriptive categories that reflect the content of the included articles. The primary author (KMK) will lead the content analysis as facilitated through NVivo software [68]. The coding will be verified by a second reviewer independently, and the coded data will be circulated amongst the entire research team. Through a series of theme discussion meetings, similarities and differences between the coded data across and within studies will be discussed [69].

The PRISMA-ScR checklist will guide the reporting of data [48].

## Limitations

Despite the systematic search strategy, we will include only English-language publications, focus our searches in the context of the healthcare transition literature. Thus, our search may inadvertently miss other literature, such as articles not published in English. In conducting this systematic literature review, we focused our search on English-language publications within the context of the healthcare transition literature. This decision was influenced by practical considerations related to our research team’s language proficiency and available resources. Given that the authors do not have proficiency in languages other than English and lack access to translation service, we made a deliberate choice to include only English-language publications to ensure the consistency and comparability of the selected studies and to facilitate the review process within our constraints.

## Discussion

This article provides an overview of the methods to conduct a scoping review exploring methods to include older adults with various disabilities within the existing transitional care literature. The results of the proposed scoping review are relevant to any researcher interested in developing, implementing and evaluating transitional care interventions for older adults with various forms of disabilities. By working to understand better innovative methods for involving diverse older adults with disabilities in evaluating transitional care interventions across places of care, we seek to help ensure that future interventions best meet the needs of the patients they wish to serve. Synthesizing the existing literature and understanding the diverse older adults involved in current research can also be used to improve future research processes and inform methodological insight for future studies. As such, our results will also be disseminated widely through conference presentations (e.g., the Canadian Gerontology Association Conference) and workshops and at least one peer-reviewed publication (e.g., Health & Social Care in the community).

## Supporting information

S1 Checklist *PLOS ONE* clinical studies checklist. (DOCX)

S2 Checklist PRISMA-P (Preferred Reporting Items for Systematic review and Meta-Analysis Protocols) 2015 checklist: Recommended items to address in a systematic review protocol*. (PDF)
